# Prenatal diagnosis of maternal partial trisomy 9p23p24.3 and 14q11.2q21.3 in a fetus: a case report

**DOI:** 10.1186/s13039-020-0473-x

**Published:** 2020-02-06

**Authors:** J. B. Wu, J. Sha, J. F. Zhai, Y. Liu, B. Zhang

**Affiliations:** 1 Department of Prenatal Diagnosis Medical Cente, Xuzhou Clinical School of Xuzhou Medical University, Xuzhou Central Hospital, Affiliated Hospital of Medical College of Southeast University, 199 South Jiefang Road, Xuzhou, 221009 Jiangsu China; 2 Department of Ultrasonography, Xuzhou Clinical School of Xuzhou Medical University, Xuzhou Central Hospital, Affiliated Hospital of Medical College of Southeast University, Xuzhou, China

**Keywords:** Cytogenetic analysis, Fetus, Partial 9p duplication, Partial 14q duplication, Single nucleotide polymorphism

## Abstract

**Objective:**

This study aimed to report a fetus with maternal partial trisomy 9p and 14q and the phenotype detected in ultrasound.

**Methods:**

The chromosome rearrangements in the fetus were characterized by G-banding and chromosome microarray analysis based on single nucleotide polymorphism (SNP) array of cultured amniocytes and compared with the parents’ karyotypes.

**Results:**

The fetal abnormal karyotype was 47,XY,+der(14)(9;14)(p23;q22). The SNP array revealed a duplicate 11.8-Mb 9p23-p24.3 fragment and a duplicate 29.6-Mb 14q11.2-q21.3 fragment. The peripheral blood karyotype of the mother was 46,XX,t(9;14)(p23;q22), while the father’s was normal at the level of 300~400 bands. A high-resolution karyotype analysis conformed the same abnormality of the mother at the level of 550~650 bands. These results indicated that the fetal chromosomal abnormality probably derived from the mother. The fetal nuchal translucency thickness was 3.5 mm, and the fetal heart was detected with around 1.0-mm ventricular defect by the ultrasound examination at 12-week gestation. The couple decided to terminate the pregnancy. They opted for in vitro fertilization and embryo transfer for the fourth pregnancy, which was successful.

**Conclusions:**

The SNP array combined with cytogenetic analysis was particularly effective in identifying abnormal chromosomal rearrangements. These methods combined with the existing database information and fetal ultrasonography might provide a comprehensive and efficient way for the prenatal assessment of fetal situations. Preimplantation genetic diagnosis might effectively assist those women with an adverse pregnancy history in their next pregnancy.

## Introduction

Trisomy 9p is one of the most abnormal chromosomes in newborns. However, the case of partial 9p and 14q trisomy has been reported only once to date [1]. Chromosome trisomy is usually caused by the nondisjunction of homologous chromosomes during gamete formation, especially from the balanced translocation carriers in the parents. In most cases, the trisomic segments are transmitted from the mother or father carrying balanced translocation. However, genetic changes in the embryo often result in clinical phenotypic changes. The degree of phenotype is closely related to the extension of chromosome duplication or deletion segments. In other words, the phenotypes are connected with a small supernumerary marker chromosome (sSMC) [2]. Moreover, the degree of clinical symptoms is consistent with the important functional genes in the abnormal chromosome segments. The correlation studies between phenotype and genotype indicated that the region from 9p22 to 9p24 was the minimal critical extension to result in clinical syndromes [3, 4]. The derived duplication from 14q11.2 to 14q22.3 indicated severe physical and mental retardation defects [5]. The forkhead box protein G1 (*FOXG1*) gene encompassed on 14q11.2 to 14q12 could cause severe epilepsy and developmental delay and severe speech impairment [6, 7]. This study aimed to report a fetus inheriting maternal derivative chromosome 14 with partial 9p24.3p23 and 14q11.2q21.3 duplications and abnormal phenotype, which was detected by ultrasound examination.

## Case presentation

**Fig. 1:**
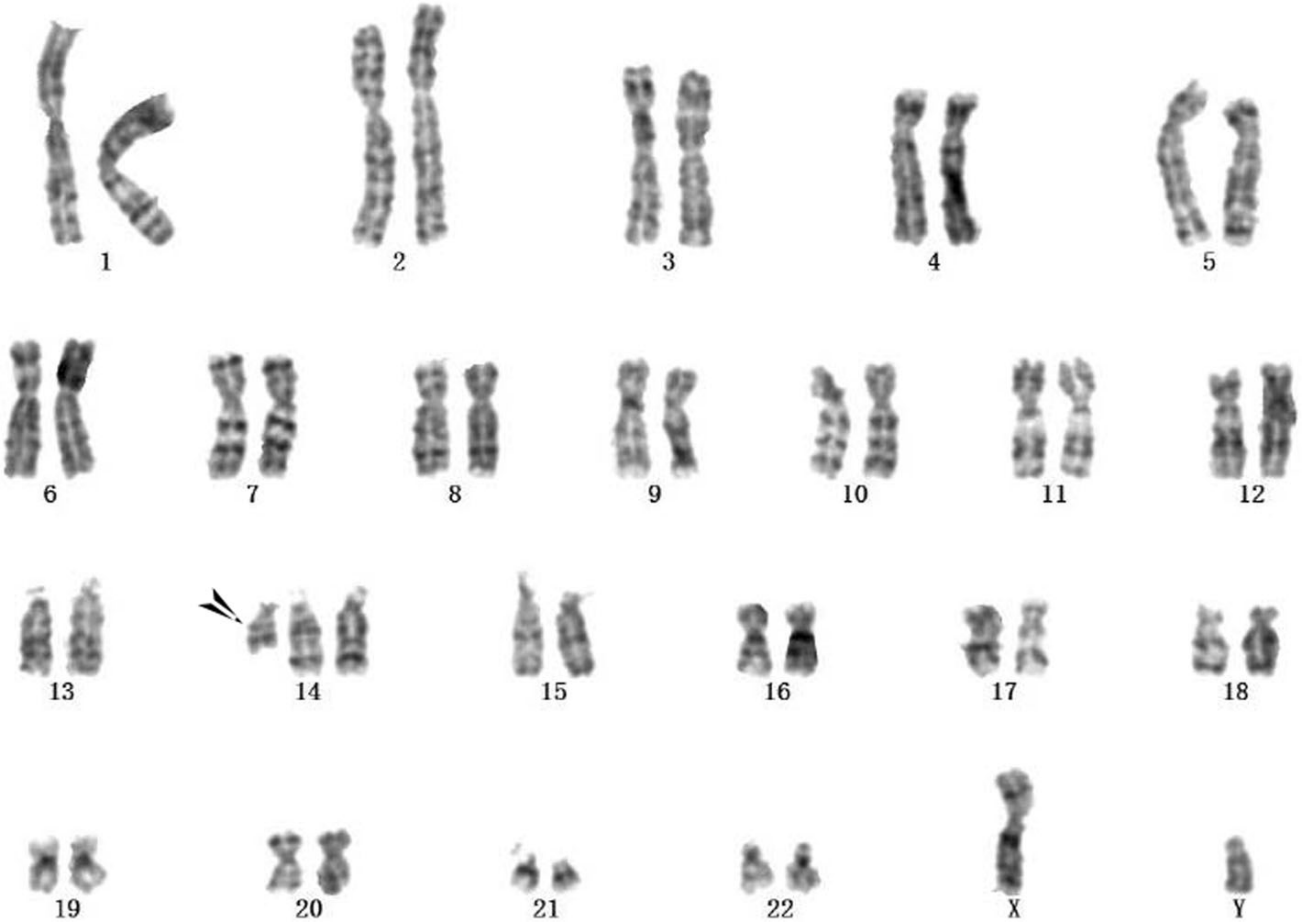
The fetal karyotype was 47,XY,+der(14)(9;14)(p23;q22)mat at the level of 300~400 bands

**Fig. 2:**
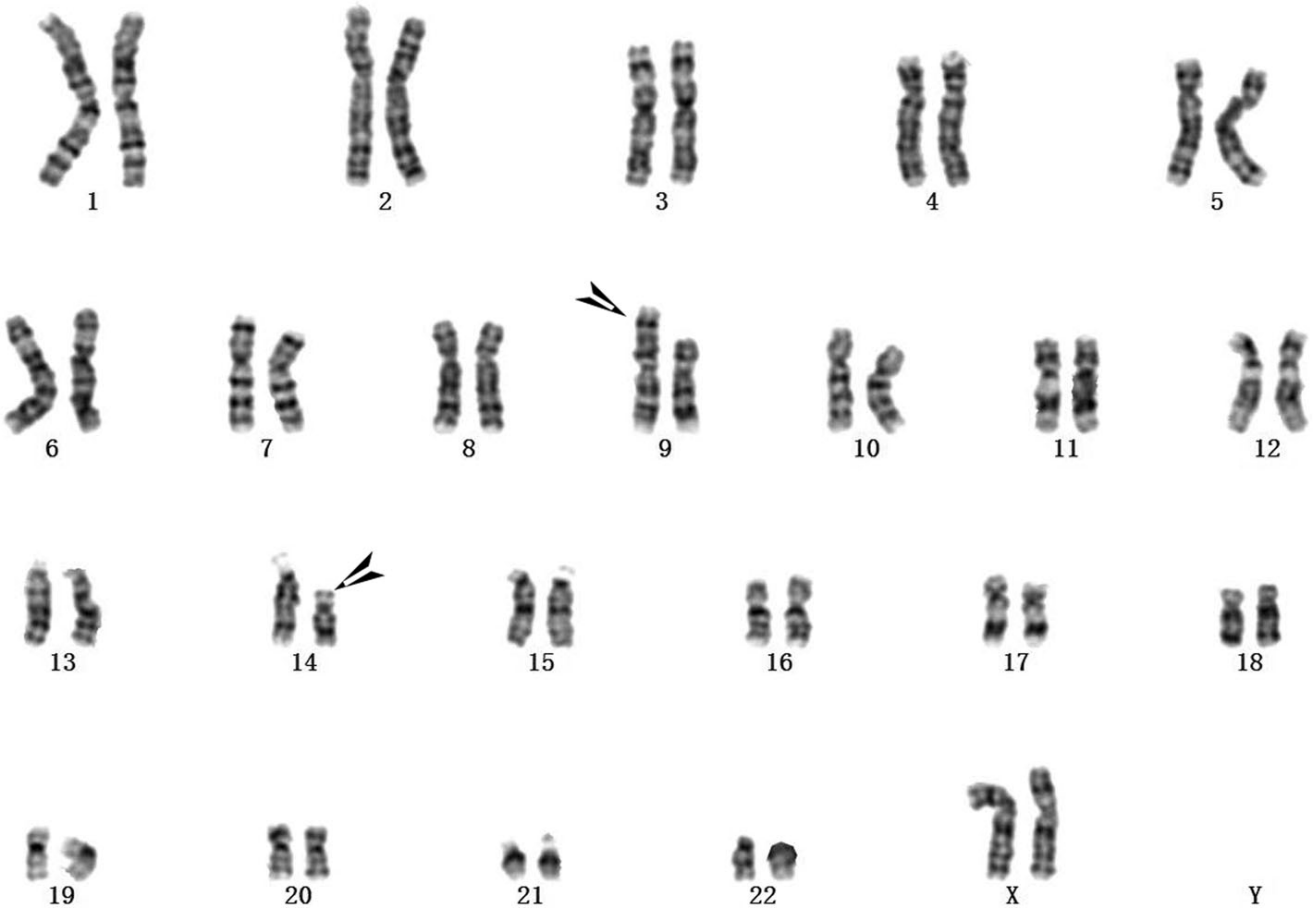
The mother’s peripheral blood karyotype was 46,XX,t(9;14)(p23;q22) at the level of 300~400 bands

**Fig. 3:**
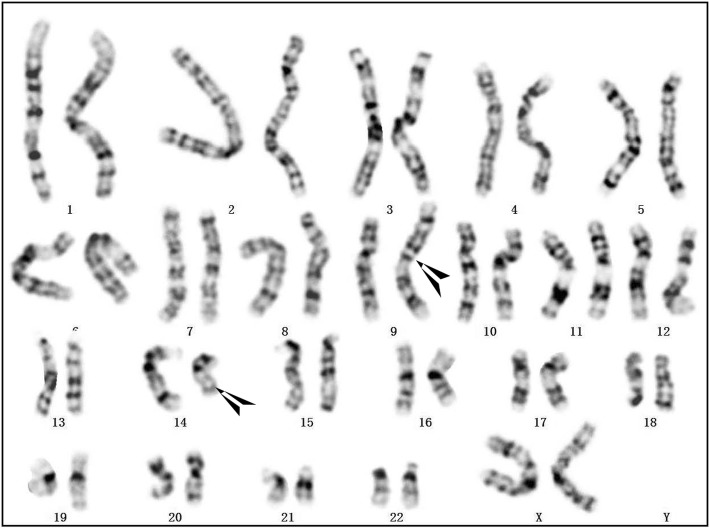
The mother’s peripheral blood karyotype was 46,XX,t(9;14)(p23;q22) at the level of 550~650 bands

**Fig. 4:**
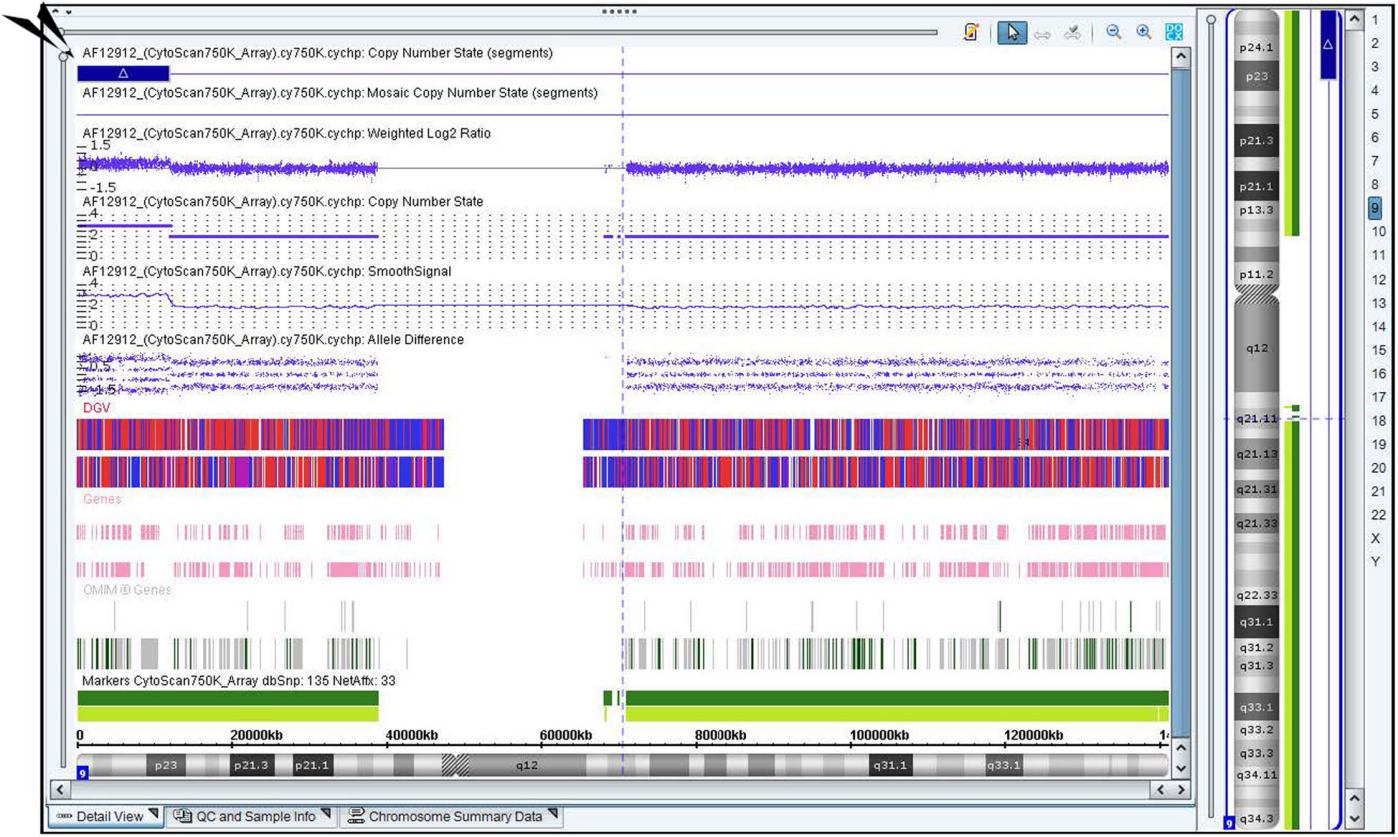
The fetus had a duplicated 11.8-Mb fragment at 9p24.3p23 in chromosome 9 (chr 9:208 454–12 064 543), containing 32 OMIM genes including GLI-similar 3 (*GLIS3*) and *SMARCA2*

**Fig. 5:**
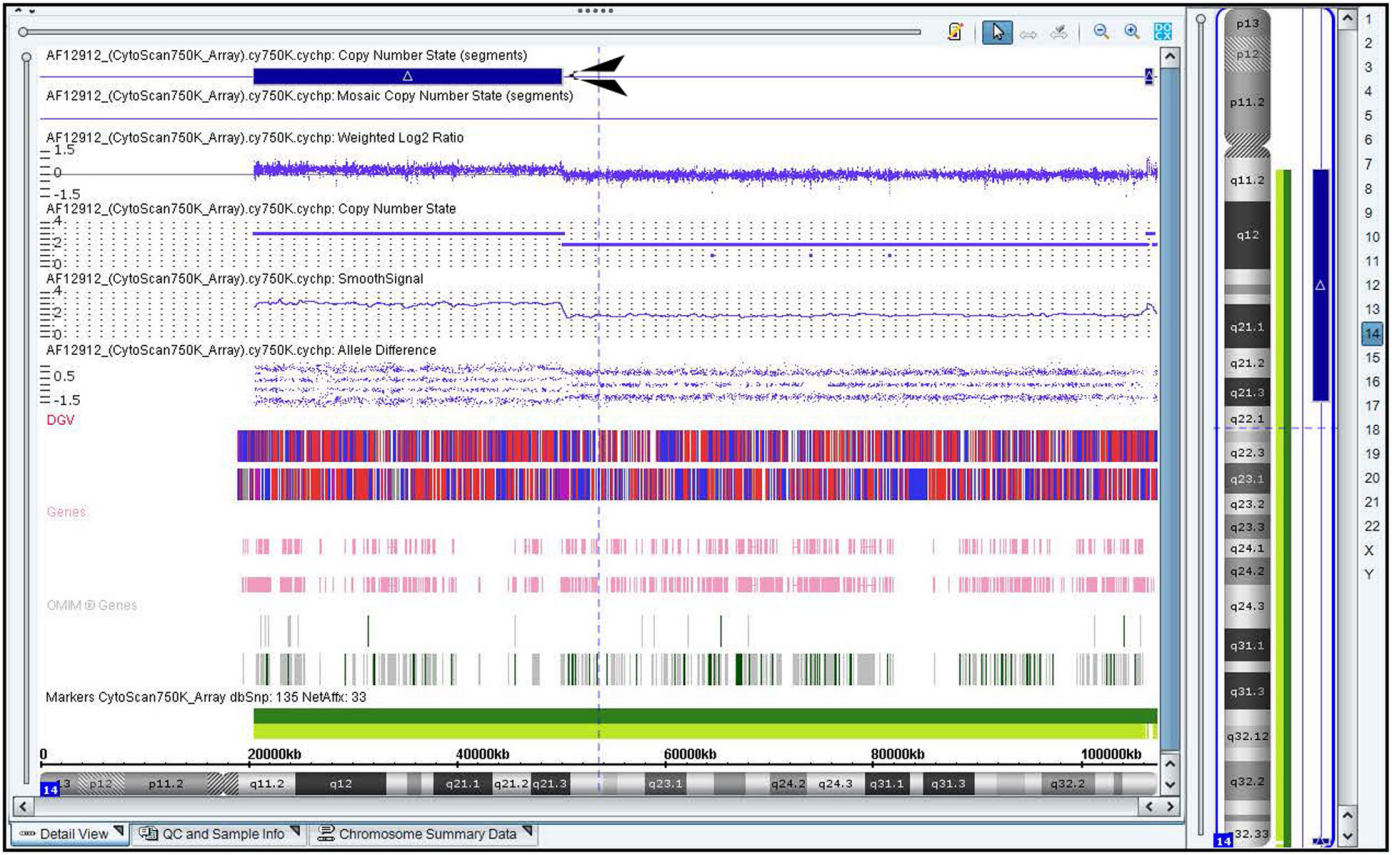
The fetus also had a duplicated 29.6-Mb fragment at 14q11.2q21.3 in chromosome 14 (chr 14: 20 516 277–50 131 335)

A 28-year-old woman who had previously experienced two early spontaneous abortions was pregnant for the third time. The couple were not consanguineous and did not have any family hereditary diseases. The woman’s last menstruation was on January 24, 2017. The nuchal translucency thickness of the fetus was 3.5 mm, and his heart had an approximately 1.0-mm ventricular defect detected in ultrasound at 12-week gestation. Amniocentesis was performed at 18-week gestation with the consent of the parents because of the two previous spontaneous abortions and the fetal structural abnormality. The fetal abnormal karyotype by G-banding was 47,XY,+der(14)(9;14)(p23;q22) at the level of 300~400 bands (Fig. 1). The SNP array revealed a duplicate 11.8-Mb fragment and a duplicate 29.6-Mb fragment with the suspended amniotic cells (Figs. 4 and 5). The couple underwent karyotype analysis to further identify the source of fetal chromosomal abnormalities and the arrangement of the cytological changes.. The results showed the same chromosomal abnormalities in the mother (Fig. 2), but no abnormality in the father. A high-resolution karyotype analysis identified the same abnormal karyotype of the mother at the level of 550~650 bands once more (Fig. 3). Combined with the CMA results, this study concluded that the fetus had an extra derivative materal chromosome with partial 9p and 14p duplication. The couple decided to terminate the pregnancy at 24-week gestation after they were informed of the possible serious consequences. A 724 g fetus was delivered with low-set ears. They selected preimplantation genetic diagnosis (PGD) to assist the next pregnancy.

### Cytogenetic and SNP array analyses

Amniocytes and peripheral blood lymphocytes of the couple were routinely collected, cultured, and harvested. G-banding was performed, followed by conventional cytogenetic analysis. Then 47,XY,+der(14)(9;14)(p23;q22) of the fetus and 46,XX,t(9;14)(p23;q22) of the mother were found according to the international system for human cytogenomic nomenclature (ISCN) 2016. Further, a high-resolution chromosome analysis of the mother’s peripheral blood was performed. The SNP array of suspended cultured amniocytes was conducted using the SNP array CytoScan 750 K probes (Affymetrix, CA, USA). The Chromosome Analysis Suite software (ChAS) was adopted for data analysis, and the results were analyzed using multiple databases, such as Online Mendelian Inheritance in Man (OMIM) and Genome.

## Results

The fetal karyotype was 47,XY,+der(14)(9;14)(p23;q22)mat at the level of 300~400 bands (Fig. 1). The mother’s chromosome was the same as that of the fetus at the level of both 300~400 (Fig. 2) and 550~650 bands (Fig. 3). However, the karyotype of the father was normal. The fetus had a duplicate 11.8-Mb 9p24.3p23 fragment (arr[hg19] 9p24.3p23 (208 454–12 064 543) × 3, Fig. 4) containing 32 OMIM genes, including GLI-similar 3 (GLIS3) and SWI/SNF-related matrix associated, actin-dependent regulator of chromatin 2 (SMARCA2). The fetus also had a duplicate 29.6-Mb 14q11.2q21.3 fragment (arr[hg19] 14q11.2q21.3 (20 516 277–50 131 335) × 3, Fig. 5), containing 146 OMIM genes, including chromodomain helicase DNA-binding protein 8 (*CHD8)*, suppressor of Ty 16 homolog *(SUPT16H)*, forkhead box protein G1 (*FOXG1)* and protein kinase D1 (*PRKD1*).

### Comparison with the literature

We compared the clinical phenotypes of the fetus with those previously reported cases with duplication of chromosome 9 and 14 (Tables 1 and 2). Table 1 gave an overview clinical abnormal performance of the patients with partial trisomy 9p at least overlapping with duplicated segment in our index fetus. At the same time, we listed clinical manifestations of the patients with partial trisomy 14q on the Table 2. There was mostly apparent consistency in the facial and limb anomalies and developmental delay and mental retardation in the patients with partial trisomy 9p and (or) 14q which might vary in degree listed in the tables above. Less common findings were congenital heart defects. A female infant born at 35 weeks gestation with duplicated 9p13p24.3 and 14p13q22 showed craniofacial anomalies and limbs abnormalities and a patent ductus arteriosus [1].

**Table 1:** Comparison of clinical phenotypes of the index patients with a partial trisomy 9p listed in order of the duplicated area of chromosome 9

Ref	patients	duplicated fragment	Sex	Age at examination	Delayed development	Mental retardation	Typical face^a^	Congenital heart defect	Typical limbs^b^	size of the duplication	other means of the analysis
Cuoco C,et al. [8]	case2	p12~p24	F	15 years	+	+	+		+		banding (QFQ, RBA, C)
	case5	p12~p24	F	26 years	+		+		+		banding (QFQ), DaDAPI, C)
Motegi T, et al. [9]	case	p12~p24	M	3 months	+		+		+		
Tsezou A, et al. [10]	case1	p12~p24	M	10 months	+		+		+		FISH/CGH
	case2		M	6 months	+		+		+		FISH/CGH
Park IY, et al. [11]	case	p13~p24	M	newborn	+		+		+		FISH
Phelan MC, et al. [12]	case	p13~p24	M	5.5 months	+	moderate	+				
Temtamy SA et al. [13]	case1	9p	F	4 years and 10 months	+	severe	+		+		FISH
	case2	p21~p24	M	8 years	+	severe	+		+		FISH
	case3	p21~p24	M	7 years and 5 months	+	+	+		+		FISH
	case4	9p	F	1 year and 7 months	+	+	+		+		FISH
	case5	9p	F	5 years	+	+	+		+		FISH
Haddad BR, et al. [4]	case1	p22~p24	F	9 years		low normal	+		+		FISH
	case2		M	44 years		low normal	+	+	+		FISH
Achkar WA, et al. [14]	case	p22~p24.2	F	20 years	+	+	+		+		FISH/aMCB
Our patient	case	p23~p24.3	M (fetus)	24 weeks			+	+		11.8 Mb	SNP-array
Guilherme RS, et al. [15]	case1	p24.3~q21.11	M	17 years	+	+	+		+	69.9 Mb	FISH/SNP-array
	case2	p24.3~q21.11	F	6 years	+	+	+		+	69.9 Mb	FISH/SNP-array
	case3	p24.3~q13	F	6 years and 9 months	+	+	+		+	68.2 Mb	FISH/SNP-array
	case4	p24.3~q13	M	17 years	+	+	+		+	67.9 Mb	FISH/SNP-array
	case5	p24.3~q13	F	6 years	+	+	+		+	67.9 Mb	FISH/SNP-array

^a^ Typical face indicates microcephaly, large anterior fontanelle, bulbous nose with nasal bridge, ptosis, deep set eyes, narrow palpebral fissures, apparent hypertelorism, low set ears, short philtrum, downturned mouth, jaw hypoplasia, short and wide neck. ^b^ Typical limb includes cubitus valgus, bilateral clinodactyly of the fifth finger, brachydactyly, short hands and feet, flat feet, clubfeet

**Table 2:** Comparison of clinical phenotypes of the index patients with a partial trisomy 14q listed in order of the duplicated area of chromosome 14

Ref	duplicated fragment	Sex	Age at examination	Delayed development	Mental retardation	Typical face^a^	Congenital heart defect	Typical limbs^b^	size of the duplication	other means of the analysis
Coco R, et al. [16]	Pter→q12~13	F	1 year	+		+				
Simpson J, et al. [17]	Pter→q12~13	F	8 months	+		+		+		
Laurent C, et al. [18]	Pter→q12~13	F	3 months	+		+				
Fryns JP, et al. [19]	Pter→q12~13	F	7 years	+	moderate	+		+		
Fried K, et al. [20]	Pter→q21	F	19 months	+	+	+		+		
Raoul O, et al. [21]	Pter→q22~23	M	3 years	+		+		+		
Turleau C, et al. [22]	Pter→q22~23	M	1.5 years	+		+				
Allderdice PW, et al. [23]	Pter→q22~23	F	4 years	+	+	+				
Muldal S, et al. [24]	Pter→q22~23	F	16 years	+	+	+		+		
Fawcett WA, et al. [25]	Pter→q22~23	F	6 months	+		+				
Yeatman GW, et al. [26]	Pter→q22~23	F	12 years	+		+				
Reiss JA, et al. [27]	Pter→q24	M	10 months	+		+	+	+		
Lopez Pajares I, et al. [28]	Pter→q24	F	2 months			+	+	+		Q-banding
Short EM, et al. [29]	Pter→q24	M	3 days	+		+	+	+		
Monfort S, et al. [30]	centromere to 14q11.2	M	14 years	+	+	+			5.38 Mb	MLPA/aCGH
Smyk M, et al. [31]	14q11.2	M	7 years		+	+			445 kb	CGH
Our patient	q11.2 → q21.3	M (fetus)	24 weeks			+	+		29.6 Mb	SNP-array
Wannenmacher B, et al. [5]	q11.2 → q22.3	M	33 years	+	+	+		+		STR/FISH
Ito M, et al. [32]	q13 → q22	M	7 years	+		+				

^a^ and ^b^ stand for the same contents listed in the Table 1

### Follow-up outcomes

In early March 2018, the couple underwent one cycle of in vitro fertilization (IVF) and embryo transfer for the fourth pregnancy and selected the PGD pregnancy procedure in the People’s Hospital of Jiangsu Province. Subsequently, an amniocentesis chromosome examination was conducted at 18-week gestation, and the karyotype of the fetus was found to be normal. Fortunately, the mother succeeded in delivering a healthy baby girl on December 11, 2018.

## Discussion

According to the principle of gamete distribution [33], the possibility of the living offspring inheriting an abnormal chromosome is 1/18 if either of a couple has a balanced translocation. The present study reported that fetal-derived chromosome 14 had partial 9p and 14q duplications. The chromosome analysis combined with the SNP array of cultured amniocyte results revealed that the fetal chromosomal abnormality probably derived from the mother. That was to say, the fetus not only inherited the normal chromosomes 9 and 14 of the parents’, but also had a derived abnormal chromosome 14 from the mother. Trisomy 9p was the fourth most frequent chromosome anomaly compatible with long-term survival in live-born infants [13, 14, 34], meanwhile trisomy 14q was not less than reported trisomy 9p in the literatures of 1970s [16–32, 35]. However, the case of partial 9p and 14q trisomy has been reported only once to date [1].

Patients with trisomy 9p are easily recognizable owing to their facial appearance. This results in complex rearrangements and the possibility that some of the duplicated genes will be dosage-sensitive, influencing the phenotype [15]. The pericentromeric region of chromosome 9 is rich in segmental duplication and low copy repeats that predispose it to nonallelic homologous recombination. With a high degree of sequence identity to sequences in 15p, 18p, and 21p, chromosome 9 is inclined to illegitimate intrachromosomal or interchromosomal recombination. The correlation studies between phenotype and genotype indicated that the region from 9p22 to 9p24 was the minimal critical extension to result in clinical syndromes [3, 4]. Patients with 9p trisomy display variable degrees of mental retardation and head and facial abnormal features, such as microcephaly with a large anterior fontanelle, micrognathia, a prominent or bulbous nose, malformed protruding ears, deep-set eyes, mild down slanting of the palpebral fissures, downturned corners of the mouth, congenital heart defects, mental retardation, and kidney and skeletal anomalies [13, 34]. A 3-year-old boy with de novo 9p24.2 to 9p23 was diagnosed with development lag and craniofacial anomalies [36]. Some studies reported that the partial duplication of 9p24.3p23 was related to microcephaly, autism, and other clinical phenotype-related diseases [4, 15, 37]. In the present study, the fetus with 9p24.3p23 contained 32 OMIM pathological genes, including *GLIS3* and *SMARCA2*. The *GLIS3* gene partially had the same chromosome segments as described in the aforementioned 3-year-old boy [36]. The fetus might be prone to neonatal diabetes complicated with congenital hypothyroidism, and have intrauterine developmental retardation during pregnancy and low-set ears and craniosynostosis after birth. *SMARCA2* gene mutations are associated with Nicolaides–Baraitser syndrome of autosomal dominant inheritance, clinical manifestations of short stature, microcephalus, dysgnosia, epilepsy, and learning disabilities. The growth and structural abnormalities were observed through an ultrasound examination. Only low-set ears and abnormal nuchal translucency thickness and heart changes of the fetus occurred during the pregnancy, but some future symptoms such as epilepsy and learning disabilities could not be detected because of the termination of pregnancy.

Another duplication of 14q11.2q21.3 of the fetus was found with 146 OMIM genes, including *CHD8*, *SUPT16H*, *FOXG1*, and *PRKD1* gene mutations closely correlated with the postnatal clinical phenotype. A 14-year-old male patient with a de novo 14q11.2 microduplication, a region significantly associated with quantitative trait loci for stature and a component of intelligence, was significantly characterized by short stature, mild mental retardation, and dysmorphic facial features [30]. A 445-kb 14q11.2 microduplication involving *CHD8* and *SUPT16H* genes causes developmental delay, intellectual disability, autism spectrum disorders, and macrocephaly, which was found in an 8-year-old boy [31]. The clinical phenotype of 14q11.2 microduplication included postpartum slow growth, microcephalus, abnormal breathing patterns, gastroesophageal reflux, dysgnosia, and agenesis of the corpus callosum [5, 30]. The *PRKD1* gene mutations are associated with autosomal dominant diseases, including congenital heart defects and ectodermal dysplasia [30, 31]. Furthermore, the thickness of the fetal nuchal translucency in the present case was 3.5 mm, and the heart had an approximately 1.0-mm ventricular defect detected during ultrasound examination at 12-week gestation, which might have been caused by the *PRKD1* gene mutation.

In addition, based on the homozygosity or heterozygosity of polymorphic alleles inherited from the parent, uniparental disomy (UPD) can be classified into isodisomy and heterodisomy. Notably, balanced familial translocationincreases the risk of fetal UPD [38]. Human chromosome 14q32.2 carries a number of imprinted genes such as delta-like non-canonical Notch ligand 1 (*DLK1*), retrotransposon-like 1 (*RTL1*), and Deiodinase, iodothyronine, type III (*DIO3*). Both paternal UPD 14 and maternal UPD 14 can cause disorders. Paternal UPD14 has been reported to be associated with Kagami-Ogata syndrome, which is characterized of polyhydramnios, developmental delay, growth retardation, abdominal defects, thoracic dysplasia with respiratory failure, and facial abnormalities [39]. Maternal UPD 14 causes Temple syndrome with multiple serious phenotypes including prenatal and postnatal growth retardation, developmental delay, joint laxity, small hands and feet, muscular hypotonia, truncal obesity, precocious puberty, and short stature [40]. The SNP array analysis from the Allele difference and BAF showed no loss of heterozygosity(LOH)in this fetus. However, heterodisomy could not be excluded despite less phenotype of this fetus in ultrasound.

The pregnancy was terminated. The couple selected one cycle of IVF and embryo transfer. Also, they chose PGD for the fourth pregnancy in early March 2018 and accepted amniocentesis during middle gestation in the People’s Hospital of Jiangsu province. Fortunately, the mother succeeded in giving birth to a healthy baby girl on December 11, 2018.

In conclusion, the SNP array combined with cytogenetic analysis might help in identifying abnormal chromosomal rearrangements. These methods combined with the existing database information and fetal ultrasonography reports may provide a comprehensive and efficient way for prenatal assessment of fetal situations. PGD effectively assists women with an adverse pregnancy history for their next pregnancy.

## Data Availability

All data generated or analysed during this study are included in this published article.

## Abbreviations

ChAS: Chromosome Analysis Suite
CMA: Chromosome microarray analysis
*DIO3*: Deiodinase, iodothyronine, type III3
*DLK1*: Delta-like non-canonical Notch ligand 1
*FOXG1*: Forkhead box protein G1
*GLIS3*: Genes including GLI-similar 3
*CHD8*: Chromodomain helicase DNA-binding protein 8
ISCN: International system for human cytogenomic nomenclature
LOH: Loss of heterozygosity
NT: Nuchal translucency
OMIM: Online Mendelian Inheritance in Man
PGD: Plantation genetic diagnosis
*PRKD1*: Protein kinase D1
*RTL1*: Retrotransposon-like 1
*SMARCA2*: *SWI/SNF* related matrix associated; actin dependent regulator of chromatin 2
SNP: Single nucleotide polymorphism
sSMC: Small supernumerary marker chromosome
*SUPT16H*: Suppressor of Ty 16 Homolog
UPD: Uniparental disomy
